# Caffeine‐mediated BDNF release regulates long‐term synaptic plasticity through activation of IRS2 signaling

**DOI:** 10.1111/adb.12433

**Published:** 2016-07-25

**Authors:** Cristina Lao‐Peregrín, Jesús Javier Ballesteros, Miriam Fernández, Alfonsa Zamora‐Moratalla, Ana Saavedra, María Gómez Lázaro, Esther Pérez‐Navarro, Deborah Burks, Eduardo D. Martín

**Affiliations:** ^1^ Laboratory of Neurophysiology and Synaptic Plasticity, Castilla‐La Mancha Science and Technology Park (PCYTCLM), Institute for Research in Neurological Disabilities (IDINE) University of Castilla‐La Mancha Spain; ^2^ Departament de Biomedicina, Facultat de Medicina Universitat de Barcelona Spain; ^3^ Institut d'Investigacions Biomèdiques August Pi i Sunyer (IDIBAPS) Spain; ^4^ Centro de Investigación Biomédica en Red (CIBER) sobre Enfermedades Neurodegenerativas (CIBERNED) Spain; ^5^ Institut de Neurociències Universitat de Barcelona Spain; ^6^ Centro de Investigación Príncipe Felipe, CIBER de Diabetes y Enfermedades Metabólicas Asociadas (CIBERDEM) Spain

**Keywords:** hippocampus, insulin receptors, NMDA‐independent synaptic plasticity, PI3K–AKT pathway, TrkB receptor

## Abstract

Caffeine has cognitive‐enhancing properties with effects on learning and memory, concentration, arousal and mood. These effects imply changes at circuital and synaptic level, but the mechanism by which caffeine modifies synaptic plasticity remains elusive. Here we report that caffeine, at concentrations representing moderate to high levels of consumption in humans, induces an NMDA receptor‐independent form of LTP (_CAF_LTP) in the CA1 region of the hippocampus by promoting calcium‐dependent secretion of BDNF, which subsequently activates TrkB‐mediated signaling required for the expression of _CAF_LTP. Our data include the novel observation that insulin receptor substrate 2 (IRS2) is phosphorylated during induction of _CAF_LTP, a process that requires cytosolic free Ca^2+^. Consistent with the involvement of IRS2 signals in caffeine‐mediated synaptic plasticity, phosphorylation of Akt (Ser473) in response to LTP induction is defective in Irs2^−/−^ mice, demonstrating that these plasticity changes are associated with downstream targets of the phosphoinositide 3‐kinase (PI3K) pathway. These findings indicate that TrkB‐IRS2 signals are essential for activation of PI3K during the induction of LTP by caffeine.

## Introduction

Caffeine, a central nervous system (CNS) stimulant (Nehlig 2010) that enhances cognitive function in both humans and experimental animals (Lieberman *et al.* 1987; Durlach 1998; Angelucci *et al.* 1999; Borota *et al.* 2014), is the most widely consumed behaviorally active substance in the world. Caffeine modulates CNS activities through several mechanisms of action including antagonism of adenosine and GABA_A_ receptors (Daly 2007), phosphodiesterase inhibition (Smellie *et al.* 1979) and sensitization of calcium‐induced calcium release through ryanodine‐sensitive channels (McPherson *et al.* 1991; Martín & Buño 2003). It has been suggested that, at the concentration typically consumed by humans, caffeine may act mainly by inhibiting adenosine receptors in the CNS (Fredholm 1995). By functioning as a competitive antagonist at A_1_ and A_2A_ adenosine receptors, it leads to diminished endogenous adenosinergic tone (Fredholm *et al.* 1999; Chen *et al.* 2010).

Several lines of evidence indicate that caffeine modulates the brain‐derived neurotrophic factor (BDNF) system (Costa *et al.* 2008; Sallaberry *et al.* 2013). On other hand, BDNF is associated with learning and memory (Greenberg *et al.* 2009) and modulates neuronal plasticity by inducing changes in synaptic efficacy and morphology (McAllister *et al.* 1999; Thoenen 2000; Poo 2001). In particular, activity‐dependent secretion of BDNF plays a critical role in long‐term potentiation (LTP; Aicardi *et al.* 2004; Lu *et al.* 2005). LTP is an activity‐dependent, persistent enhancement of synaptic strength widely studied as a cellular model for learning and memory (Malenka & Nicoll 1999). However, fundamental aspects of the mechanistic relationship between caffeine and BDNF signaling are poorly defined, particularly at the level of synaptic plasticity.

Long‐term caffeine consumption is also associated with improved peripheral glucose homeostasis (Park *et al.* 2007) and decreased risk of Type 2 diabetes (van Dam 2006). In animal models, the insulinotropic effects of caffeine administration have been linked to the signaling pathway mediated by insulin receptor substrate 2 (IRS2) (Park *et al.* 2007). IRS proteins are phosphorylated by active tyrosine kinase receptors, like insulin receptor (IR) and the insulin‐like growth factor‐1 receptor (IGF‐1R), and thereby regulate the cellular response by recruiting downstream signaling components such as phosphoinositide 3‐kinase (PI3K) and Akt (Myers *et al.* 1992; White 2006). Importantly, mice deficient for IRS2 display defects in hippocampal synaptic plasticity (Costello *et al.* 2012; Martín *et al.* 2012).

Because the neurophysiological basis for learning and memory involves long‐term modifications of synaptic efficacy in brain areas important for learning and memory such as the hippocampus (Martin *et al.* 2000), we have investigated whether caffeine modulates LTP in this brain region. To elucidate the synaptic mechanisms that underlie the functional effects of caffeine, we selected a simple and well‐known experimental system: CA3–CA1 glutamatergic synapse in the CA1 region of the hippocampus. We observed that caffeine, at concentrations representing moderate to high levels of human use (5 to 7 mg/kg, i.e. one highly‐caffeinated energy drink or three cups of coffee) induces an NMDA receptor (NMDAR)‐independent form of LTP and increases calcium‐dependent BDNF secretion, which is necessary for LTP maintenance through a TrkB‐mediated process. In addition, our studies reveal that activation of IRS2/PI3K/Akt signaling is required for the expression of caffeine‐mediated LTP in the CA1 region of the hippocampus.

## Materials and Methods

### Animals

All experiments were performed in compliance with national regulations regarding animal welfare and were approved by the institutional committee for ethics and animal use. Experiments were carried out in adult males and females NMRI mice (3–4 months of age). Male BDNF^+/−^ mice and their wild‐type littermates (3 months of age; B6CBA background) were obtained and genotyped as previously reported (Ernfors *et al.* 1994; Giralt *et al.* 2009). The generation and routine genotyping of Irs2^+/+^ and Irs2‐deficient mice (3 months of age; C57Bl/6 background) have been described previously (Withers *et al.* 1998; Burks *et al.* 2000). Animals were assigned randomly to the different experimental groups.

### Electrophysiology

Detailed methods of most of the procedures have been described previously (Martín & Buño 2003, 2005). Briefly, transverse slices (400 µm thick) from brain mice were cut with a vibratome (VT1200S, Leica Microsystems, Nussloch, Deutschland) and incubated for >1 hour at room temperature (21–24°C) in artificial cerebrospinal fluid (aCSF) that contained (in mM) 124 NaCl, 2.69 KCl, 1.25 KH_2_PO_4_, 2 MgSO_4_, 26 NaHCO_3_, 2 CaCl_2_ and 10 glucose and gassed with a 95 percent O_2_, 5 percent CO_2_ mixture at pH 7.2–7.4. Individual slices were transferred to an immersion recording chamber where they were completely submerged by oxygenate aCSF flowing at 2 ml/min (30 ± 2°C). Field excitatory postsynaptic potentials (fEPSPs) were recorded via a carbon fiber microelectrode (Carbostar‐1, Kation Scientific, Minneapolis, USA) placed in the stratum radiatum of the CA1 pyramidal layer. Evoked fEPSPs were elicited by stimulation of the Schaffer collateral fibers (SCs) with an extracellular bipolar tungsten electrode via a 2100 isolated pulse stimulator (A‐M Systems, Inc., Carlsborg, WA, USA) that was set to deliver monophasic currents of 50‐µs duration. At the beginning of each experiment, basal synaptic transmission was analyzed by applying isolating stimuli of increasing intensity to reach a maximal fPEPS response. For LTP experiments, the stimulus intensity was adjusted to elicit 50 percent of the maximum response signal and kept constant throughout the experiment. Data were stored in a Pentium‐based PC through an acquisition system PowerLab 4/26 (AD Instruments, Bella Vista, Australia) and the software Scope (AD Instruments) was used to display fEPSPs and measurements of fEPSPs slopes. The recording was discarded if the baseline fEPSP slope was not stable for 30 min and/or if the reproducible fEPSP amplitude change > 20 percent from initial values. After recording stable baseline responses for 30 min, LTP was elicited by a short‐perfusion (5 min) with caffeine (usually 100 μM) and potentiation was measured for 1 hour after LTP induction at 0.033 Hz. Under these conditions, caffeine induced a non‐decremental potentiation in 65.57 percent of all cases tested. We defined positive LTP as an increase in the fEPSP slope of at least 15 percent with respect to the baseline that was maintained 60 min after initiation of caffeine perfusion. However, all slices (potentiated and no potentiated) were included in the analysis of data to compare statistically different treatments. High frequency stimulation (HFS) was induced by applying 4 trains (1 s at 100 Hz) spaced at 20 s in order to elicit a LTP superimposed on the LTP evoked by caffeine. Changes in the fEPSP slope were calculated in relation to the baseline fEPSP responses during the last 10 min before caffeine perfusion. The time course of the fEPSP response was then normalized to this baseline. The pre‐ or postsynaptic origin of the observed modification of fEPSP slopes by caffeine was tested by estimating changes in the paired pulse facilitation (PPF), which are considered of presynaptic origin (Creager *et al.* 1980; Martín & Buño 2003, 2005). PPF was studied by the application of pairs of stimuli at different interpulse intervals (15 to 400 ms) every 30 s. The stimulus intensity was adjusted to elicit 30–50 percent of the maximum response signal (mean stimulation strength of 3.5 ± 1.5 V). PPF index was calculated from the expression (R2 − R1) / R1, where R1 and R2 were the slopes of the first and second fEPSPs, respectively.

### Drugs

Ryanodine, 8‐Cyclopentyl‐1,3‐dipropylxanthine (DPCPX), thapsigargin, suramin, and D (−)‐2‐amino‐5‐phosphonopentanoic acid (AP5) were purchased from Tocris Cookson (Bristol, UK). All other drugs were purchased from Sigma (St Louis, MO, USA). Drugs were stored as frozen concentrated stock solutions and dissolved in oxygenated aCSF at the desired concentration immediately before use. If not otherwise mentioned, drugs were added 20 min before caffeine perfusion and were present in the chamber for the entire recording period.

### Immunoprecipitation and western blotting

Hippocampal slices were prepared for electrophysiology as described above. Prior to caffeine treatment, slices were collected and processed as non‐treated basal controls. Following caffeine perfusion, hippocampal slices were collected at 5, 15, 30 and 60 min and frozen immediately. Tissue was lysed in RIPA buffer: 150 mM NaCl, 50 mM Tris, 1 mM EDTA, 1 mM EGTA, 0.1 percent SDS, 0.5 percent sodium deoxycholate, 1 percent Igepal CA‐630, 1 mM Na_3_VO_4_, 1 mM NaF, 2 mM phenylmethylsulfonyl fluoride (PMSF) and 10 µg/ml each aprotinin and leupeptin. Homogenates were clarified by centrifugation at 12 000 ×*g* for 10 min at 4°C. Protein concentration was quantitated using the Bio‐Rad DC protein assay (Bio‐Rad, Hercules, CA). Immunoprecipitations were performed using 500 µg of total protein. The immune complexes were collected on protein A‐agarose beads and subjected to SDS‐PAGE. Gels were transferred to polyvinylidene difluoride (PVDF) membranes (Amersham Biosciences, Little Chalfont, UK) and incubated with the following primary antibodies: anti‐phospho‐Akt (Ser473) (1:1000; #9271), anti‐Akt (pan) (40D4) (1:1000; #2920), anti‐IR (L55B10) (1:100; #3020), anti‐IGF‐1R β (1:1000; #3027), anti‐P‐GSK3beta (Ser9) (1:2000; #9336) and anti‐GSK3beta (27C10) (1:2000; #9315) were purchased from Cell Signaling (Danvers, MA, USA). Anti‐phosphotyrosine, clone 4G10® (1:1000; #05‐321), anti‐IRS2 (1:1000; #MABS15) and anti‐α‐tubulin (1:5000; #CP06) were purchased from Millipore (Molsheim, France). Anti‐TrkB (1:1000; #AF1494) was purchased from R&D Systems (Minneapolis, MN, USA). Anti‐IRS1 (1:1000; #sc‐559) were purchased from Santa Cruz Biotechnology (Heidelberg, Germany). Anti‐rabbit HRP (1:5000; #111‐035‐003) and anti‐mouse HRP (1:5000; #115‐035‐003) (Jackson ImmunoResearch Laboratories, Pennsylvania, USA), were used as secondary antibodies. Chemiluminescence detection was performed with an enhanced chemiluminescence detection kit (Amersham ECL RPN 2209 Kit) and Western blot signals were analyzed with an ImageQuant^TM^ LAS 4000 laser scanning and imaging system (Fujifilm, Tokyo, Japan). Immunoreactive bands were quantified using the Fujifilm Imager software (Fujifilm Medical Systems USA, Inc., Stamford, CT). Values reported from Western blots were obtained by density analysis and expressed as the ratio protein of interest/tubulin expression. For the quantification of phosphorylated proteins, band intensities were normalized to the band representing the total amount of corresponding protein and expressed as percentage of basal phosphorylated protein levels.

### Measurement of BDNF

BDNF levels in the perfusion medium were quantified by ELISA (Mouse BDNF ELISA Kit, Biosensis, Thebarton, Australia) as previously described (Aicardi *et al.* 2004). Briefly, 96‐well plates were coated with anti‐BDNF monoclonal antibody overnight at 4°C. Next, the plates were washed and blocked, followed by 2‐hour incubation with the samples. The assay was performed according to the manufacturer, except that the concentration of the anti‐BDNF monoclonal and anti‐human BDNF polyclonal antibody was 2 µl/ml and 4 µl/ml, respectively, and the dilution of the anti‐IgY‐HRP antibody was 5 µl/ml. BDNF samples used to generate the standard curves were incubated in the same plate. The plates were extensively washed, and the anti‐human BDNF polyclonal antibody was applied, followed by subsequent steps according to the protocol of the manufacturer. Absorbance values were read at 450 nm in a plate reader (Bio‐Rad).

### Data analyses

Randomization of slices was ensured by arbitrarily selecting slices for assessment after incubation, based on a pre‐established working protocol. Positive control LTP measurements were performed alternately with experimental tests (i.e. caffeine perfusion with and without treatment, as well as alternate mouse genotype). The slices were discarded if the recording of baseline fEPSP slope was not stable for 30 min and/or if the reproducible fEPSP amplitude varied >20 percent from initial values. Because continuous observation by the operator is required throughout the time‐course to guarantee correct acquisition of recording signals, files were keyed and stored by a different lab member to blind investigator during data analysis. In each experiment, ***N*** represents the number of animals whereas ***n*** represents the number of slices (all, potentiated and no potentiated). We attempted to reduce animal use, but included sufficient samples to permit robust analysis. All data are expressed as mean ± standard error of the mean (SEM). Statistical analyses were performed by using the unpaired Student's *t*‐test (95 percent confidence) or the one‐ or two‐way ANOVA with Dunnett's or Bonferroni's *post hoc* test, as appropriate and indicated in the figure legends. Values of *P* < 0.05 were considered as statistically significant.

## Results

### Caffeine‐mediated LTP is NMDAR independent and requires intracellular Ca^2+^

**Figure 1:**
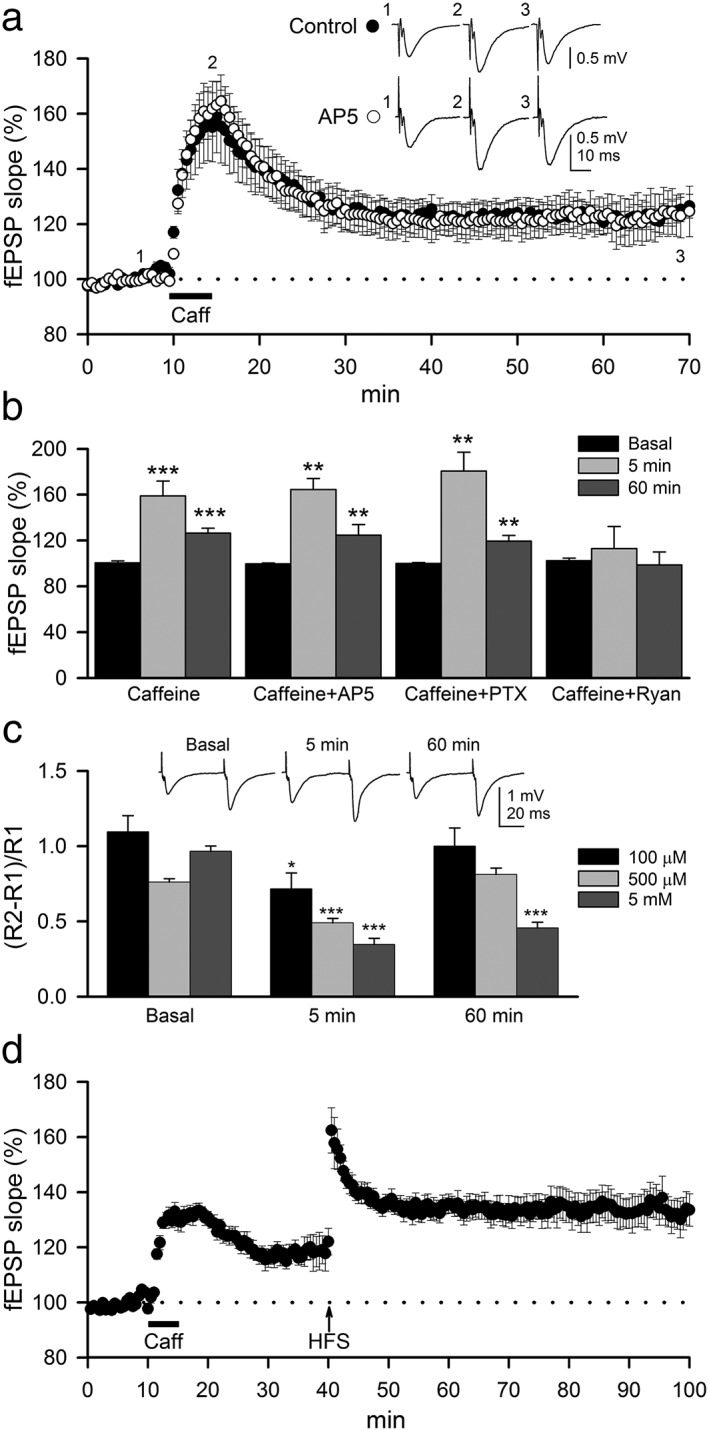
Caffeine induces an NMDAR‐independent LTP that does not occlude the classical LTP evoked by high frequency stimulation. (A) Summary of time‐course of mean fEPSPs slope in basal conditions and following bath application of 100‐μM caffeine (5 min, bar) in control slices (filled circles, *n* = 12; *N* = 7) and in the presence of 50‐μM AP5 (empty circles, *n* = 7; *N* = 3). Traces inset in the plots represent fEPSPs averages recorded during periods indicated by corresponding numbers in the graph. (B) Summary data showing mean fEPSP slopes in hippocampal slices before (Basal) and after (5 and 60 min) application of caffeine in control condition (*n* = 12; *N* = 7; same as (A)) and in the presence of 50‐μM AP5 (*n* = 7; *N* = 3), 50‐μM picrotoxin (PTX, *n* = 7; *N* = 3) and 20‐μM ryanodine (Ryan, *n* = 7; *N* = 3). (C) Mean average of paired‐pulse facilitation (PPF) at interstimulus interval of 50 ms in basal condition and after perfusion with caffeine at different concentrations (100 μM: *n* = 12; *N* = 6; 500 μM: *n* = 14; *N* = 6; 5 mM: *n* = 7; *N* = 3). Insets of tracings in the plots represent fEPSPs averages recorded in basal conditions and after 5 and 60 min of 100‐μM caffeine perfusion. (D) Summary data showing LTP induced by 100‐μM caffeine (5 min, bar) following by high frequency stimulation of SC (HFS ↑) after 30 min (filled circles, *n* = 11; *N* = 4). Significant differences with respect to basal state were established with Student's *t*‐test at *P < 0.05, **P < 0.01 and ***P < 0.001

To perform this series of experiments, we employed an intermediate concentration of caffeine (100 μM) representing moderate to high levels of human use (Lelo *et al.* 1986; Fredholm *et al.* 1999). A single dose of bath‐perfused caffeine (100 μM, 5 min) induced a marked potentiation of the fEPSP slope (158.7 ± 12.9 percent; 5 min after caffeine; *n* = 12; *N* = 6; Fig. 1A, B), termed _CAF_LTP, that was sustained 60 min after perfusion of caffeine (126.5 ± 4.3 percent; *n* = 12; *N* = 6; Fig. 1A, B). Blocking NMDARs with AP5 (50 μM), which prevents the classical LTP (Nicoll & Malenka 1999), did not modify _CAF_LTP in any of the slices tested (Fig. 1A, B; *n* = 7; *N* = 3) ruling out a contribution of postsynaptic NMDARs to _CAF_LTP, in agreement with previous findings (Martín & Buño 2003, 2005). The influence of GABA_A_‐mediated inhibitory synaptic transmission was also discarded because perfusion with caffeine in the presence of the GABA_A_ receptor antagonist picrotoxin (PTX, 50 μM) did not alter _CAF_LTP (Fig. 1B; *n* = 7; *N* = 3). Conversely, treatment with ryanodine (20 μM) inhibited _CAF_LTP in all slices tested (Fig. 1B; *n* = 7; *N* = 3) without modifying basal synaptic transmission or PPF (Fig. S1). These data indicate that caffeine‐induced Ca^2+^ release from ryanodine‐sensitive stores plays a major role in the induction of the _CAF_LTP (Martín & Buño 2003, 2005).

To determine whether the effects of caffeine on synaptic transmission were exerted pre or postsynaptically, we estimated changes in PPF (see Methods), which are considered of presynaptic origin (Creager *et al.* 1980; Martín & Buño 2003). _CAF_LTP was paralleled by a significant concentration‐dependent reduction of PPF during caffeine perfusion (100 μM: 0.72 ± 0.10; *P* < 0.05; *n* = 12; *N* = 6; 500 μM: 0.49 ± 0.03; *P* < 0.001; *n* = 14; *N* = 6; 5 mM: 0.34 ± 0.04; *P* < 0.001; *n* = 7; *N* = 3; Fig. 1C). These values returned to baseline at 60 min in samples treated with the moderate and high concentration (100 and 500 μM), but remained reduced at very high concentrations (5 mM; 0.45 ± 0.04; *P* < 0.001; *n* = 7; *N* = 3; Fig. 1C), in agreement with previous results (Martín & Buño 2003, 2005). These data suggest that presynaptic mechanisms are involved in the induction of LTP at moderate to high concentrations of caffeine, whereas postsynaptic mechanisms appear to play a major role in the maintenance of _CAF_LTP.

To investigate whether _CAF_LTP and classical LTP induced by electrically stimulation of SCs share similar cellular mechanisms, we next determined if _CAF_LTP can occlude LTP induced by HFS. After stabilization of _CAF_LTP (30 min), we applied 4 trains (1 s at 100 Hz) spaced at 20 s (see Materials and Methods). This protocol induced a fEPSP potentiation, and the resulting LTP could be superimposed on the _CAF_LTP (Fig. 1D; *n* = 11; *N* = 7), indicating that different cellular mechanisms underlie these two forms of LTP and excluding any masking effect of caffeine on fEPSP potentiation.

### Caffeine‐induced BDNF release contributes to _CAF_LTP

**Figure 2:**
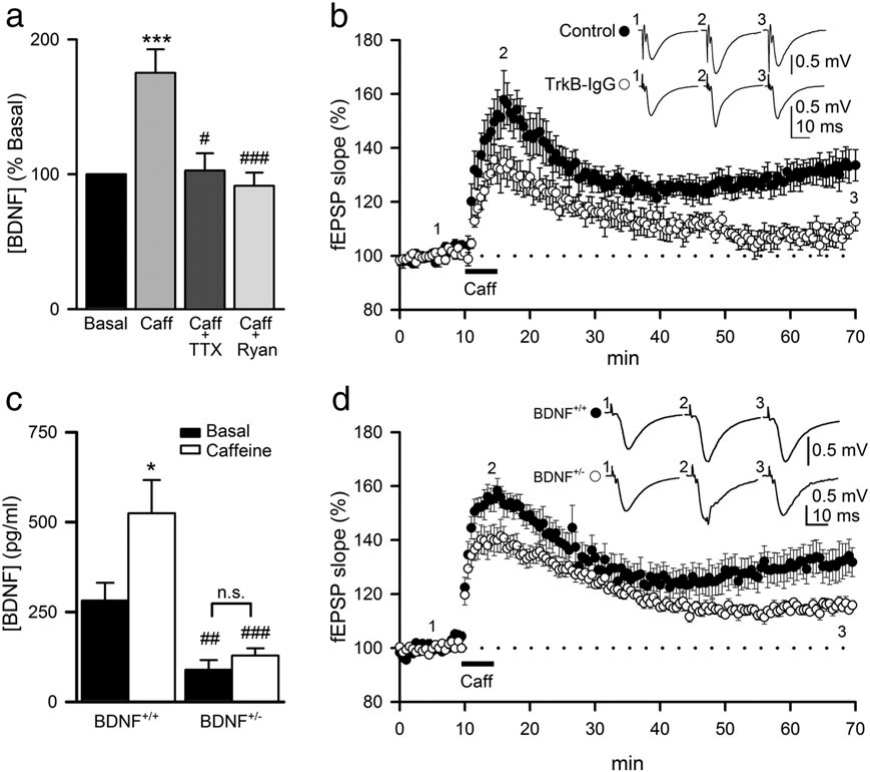
Caffeine‐induced BDNF release and TrkB activation contribute to the maintenance of _CAF_LTP. (A) Effect of caffeine on BDNF release (percent respect to basal) following 100‐μM caffeine application (5 min) in control conditions (*n* = 27; *N* = 4) and after treatment with tetrodotoxin (TTX, *n* = 12; *N* = 4) or ryanodine (Ryan, *n* = 15; *N* = 4). Significant differences were established at ***P < 0.001 versus respective control group and ^#^ *P* < 0.05 and ^###^ *P* < 0.001 versus respective caffeine group. (B) In the presence of 1 µg/ml TrkB‐IgG (empty circles, *n* = 8; *N* = 3) incubated for 1 hour before transfer of slices to the recording chamber and maintained in the perfusion medium until 15 min after caffeine application, LTP significantly decays compared to control (filled circles, *n* = 12; *N* = 7; *P* < 0.01) within 60 min. Insets of traces in the plots represent average fEPSPs recorded during periods indicated by corresponding numbers in the graph. (C) Summary data showing released BDNF levels (pg/ml) in basal condition (black bar) and after caffeine treatment (white bar) in BDNF^+/+^ (*n* = 8; *N* = 3) and BDNF^+/−^ (*n* = 8; *N* = 3) mice. Significant differences were established at *P < 0.05 versus respective basal conditions for each genotype and ^##^ *P* < 0.01 and ^###^ *P* < 0.001 between genotypes. (D) Summary of time‐course of mean fEPSPs slope in hippocampal slices from BDNF^+/+^ (filled circles; *n* = 8; *N* = 3) and BDNF^+/−^ (empty circles; *n* = 8; *N* = 3) mice in basal conditions and following application of 100‐μM caffeine. Traces inset in the plots represent fEPSPs averages recorded during periods indicated by corresponding numbers in the graph

Previous experimental work indicates that caffeine induces massive secretion of BDNF in cultured hippocampal neurons (Santi *et al.* 2006). Therefore, we next investigated whether BDNF was secreted from caffeine‐stimulated hippocampal slices. BDNF levels in the slice perfusion medium were analyzed by ELISA in fractions collected at 1‐min intervals before and after caffeine application. Caffeine applied during 5 min elicited a significant increase in BDNF levels (175.2 ± 17.4 percent; *P* < 0.001; *n* = 27; *N* = 4; Fig. 2A) that was abolished by 1‐μM tetrodotoxin (TTX; *n* = 12; *N* = 4; Fig. 2A), a selective Na^+^ channel blocker which prevents action‐potential generation, and by the blockade of Ca^2+^ release from intracellular stores with ryanodine (Ryan; *n* = 15; *N* = 3; Fig. 2A). In addition, the data suggest agreement between the probability of caffeine‐induced LTP (65.57 percent of all cases tested, see Materials and Methods) and BDNF release because for the caffeine group (*n* = 27), 18 slices showed an increase of measured BDNF above the 15 percent of basal levels (67 percent of total slices). Thus, we hypothesized that BDNF might be required to induce _CAF_LTP in hippocampal slices. To address this possibility, we first blocked the interaction of BDNF with its receptor TrkB by using a BDNF scavenger (anti‐TrkB IgG). The slices were incubated with TrkB‐IgG (1 µg/ml) for 1 hour before their transfer to the recording chamber where TrkB‐IgG was maintained in the perfusion medium until 15 min after caffeine application. Under these conditions, caffeine induced a transient increase of the fEPSP slope (135.6± 5.7 percent; 5 min after caffeine; *n* = 8; *N* = 3; Fig. 2B) followed by a gradual decline to baseline values, with potentiation of fEPSP significantly reduced as compared with control (112.6 ± 3.3 percent for TrkB‐IgG; *n* = 8; *N* = 3 versus 133.5 ± 5.8 for control; *n* = 12; *N* = 7; 60 min after caffeine; *P* < 0.01; Fig. 2B). These observations strongly suggest that TrkB activation is involved in _CAF_LTP. This finding was further supported by two additional findings: (1) caffeine‐induced BDNF release was absent in slices from BDNF^+/−^ mice (*n* = 8; *N* = 3), whose basal BDNF levels were significantly lower than in wild‐type animals (*P* < 0.01; *n* = 8; *N* = 3; Fig. 2C); (2) there was a reduction in the ability of hippocampal slices from BDNF heterozygous mice to support _CAF_LTP, with potentiation of fEPSPs being significantly minor as compared with slices from wild‐type littermates (*n* = 8; *N* = 3 of each genotype; *P* < 0.001 at 60 min after applying caffeine; Fig. 2D). Taken together, these results indicate that caffeine evokes an increase of calcium‐dependent BDNF secretion during _CAF_LTP that is necessary for NMDAR‐independent LTP maintenance through a TrkB‐mediated process.

### The PI3K/Akt pathway is necessary for the expression of _CAF_LTP

**Figure 3:**
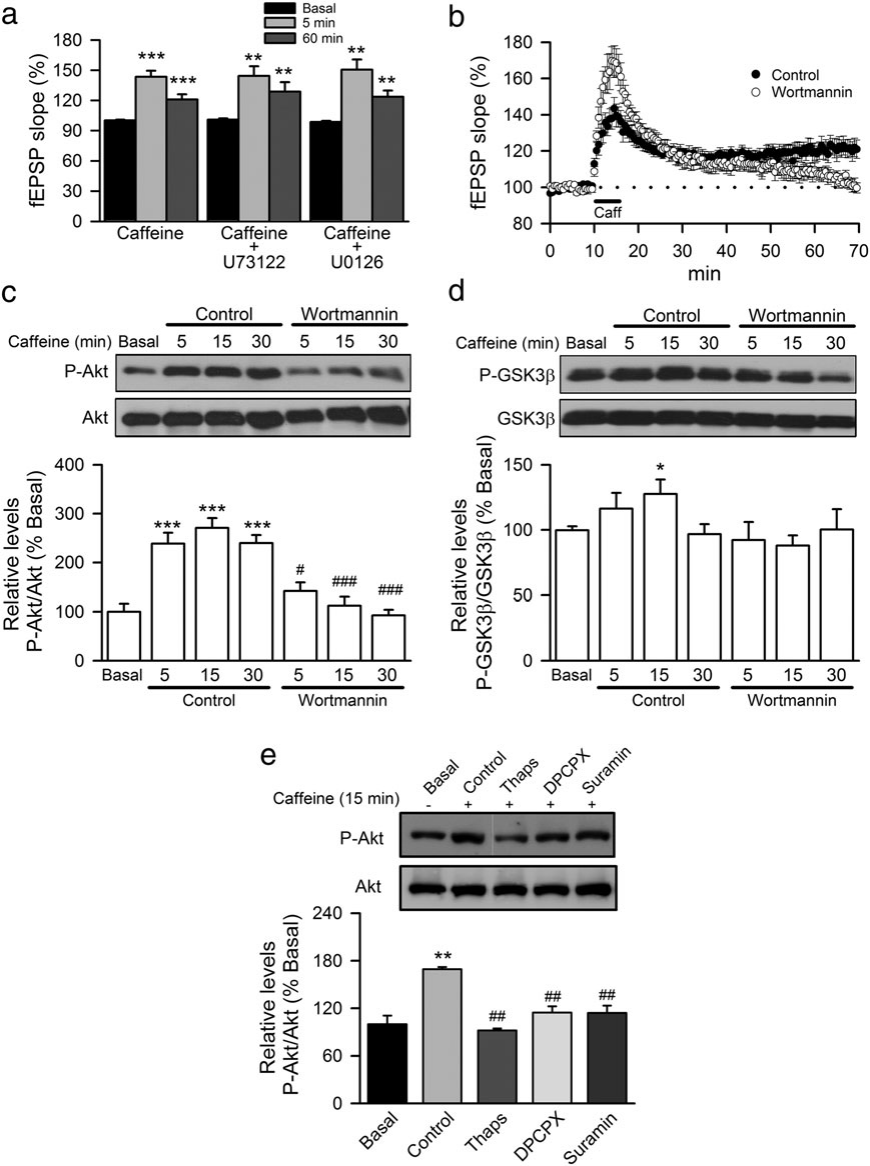
_CAF_LTP depends on PI3K/Akt pathway signaling. (A) Summary data showing mean fEPSP slopes in hippocampal slices before and after (5 and 60 min) application of caffeine in control condition (*n* = 12; *N* = 6) and in the presence of 10‐μM U73122 (*n* = 7; *N* = 3) or 20‐μM U0126 (*n* = 7; *N* = 3). Significant differences with respect to basal state were established with Student's *t*‐test at *P < 0.05, **P < 0.01 and ***P < 0.001. (B) Summary data showing the time‐course of mean fEPSPs slope in hippocampal slices in basal condition and after application of 100‐μM caffeine (black bar) to control slices (filled circles, *n* = 12; *N* = 6), and to slices incubated in the presence of 1‐μM wortmannin (empty circles, *n* = 9; *N* = 3). (C) Hippocampal slices were collected before (Basal) and after (5, 15 and 30 min) induction of LTP with caffeine in the absence (control) or in the presence of 1‐μM wortmannin and processed for Western blot analysis of Akt (Ser473) phosphorylation levels. Values represent mean ± SEM (*n* = 8; *N* = 3). Representative immunoblots are shown. Significant differences were established by one‐way ANOVA with Bonferroni´s *post hoc* test at ***P < 0.001 versus control slices incubated in the absence of caffeine, and ^#^ *P* < 0.05, ^###^ *P* < 0.001 as compared with slices incubated with caffeine in the absence of wortmannin. (D) Hippocampal slices were collected as in (C) and processed for Western blot analysis of GSK3β (Ser9) phosphorylation. Values represent mean ± SEM (*n* = 8; *N* = 3). Representative immunoblots are shown. Significant differences were established by one‐way ANOVA with Dunnett's *post hoc* test at *P < 0.05 versus control slices incubated in the absence of caffeine. (E) Hippocampal slices were collected before (0 min) and after (15 min) induction of _CAF_LTP in the absence (Control) or in the presence of 1‐μM thapsigargin (Thaps), 100‐nM DPCPX or 10‐μM suramin and processed for Western blot analysis of Akt (Ser473) phosphorylation levels. Values represent mean ± SEM (*n* = 8; *N* = 3 for each treatment). Representative immunoblots are shown. Significant differences were established by one‐way ANOVA with Bonferroni's *post hoc* test at **P < 0.01 versus control slices incubated in the absence of caffeine, and ##*P* < 0.01 as compared with slices incubated with caffeine in the presence of inhibitors

The binding of BDNF to TrkB activates several well‐known intracellular signaling cascades (Kaplan & Miller 2000). To identify the pathway(s) responsible for BDNF‐mediated _CAF_LTP, we inhibited three downstream BDNF/TrkB signaling pathways involved in hippocampal LTP: the phospholipase Cγ (PLCγ) pathway, the Ras‐mitogen‐activated protein kinase (MAPK) pathway and the PI3K pathway (English & Sweatt 1996; Kanterewicz *et al.* 2000; Man *et al.* 2003; Wang *et al.* 2003). Inhibition of PLCγ (10‐μM U73122; *n* = 7; *N* = 3; Fig. 3A) or MAPK (20‐μM U0126; *n* = 7; *N* = 3; Fig. 3A) was ineffective in blocking _CAF_LTP. In contrast, treatment with the PI3K inhibitor wortmannin (1 μM; *n* = 9; *N* = 3) completely abolished the maintenance of _CAF_LTP (Fig. 3B). Consistent with this result, we observed that the levels of phosphorylated Akt (Ser473) increased after caffeine treatment (*n* = 8; *N* = 3 for each experimental condition; *P* < 0.001; Fig. 3C), and that this increase was prevented by the PI3K inhibitor wortmannin (1 μM; *n* = 8; *N* = 3 for each experimental condition; Fig. 3C). In this line, the levels of phosphorylated GSK3β (Ser9), a downstream target of Akt, also increase significantly 15 min after caffeine, an effect prevented by wortmannin (*n* = 8; *N* = 3; *P* < 0.05; Fig. 3D). Depletion of the internal Ca^2+^ stores with 1‐μM thapsigargin also blocked the increase in the levels of phosphorylated Akt (Ser4739) induced by caffeine (*n* = 6; *N* = 2; Fig. 3E). Collectively, these results suggest that PI3K/Akt signaling is necessary for the maintenance of _CAF_LTP.

Previous experimental work has suggested that A1 adenosine receptors and P2 purinergic receptors are required for _CAF_LTP (Martín & Buño 2003). Therefore, we next investigated whether the PI3K/Akt pathway is modulated by purinergic signaling. The caffeine‐mediated stimulation of Akt phosphorylation was significantly reduced in the presence of the A1 adenosine receptor antagonist DPCPX (100 nM; *n* = 6; *N* = 2; *P* < 0.01; Fig. 3E) and the non‐selective P2 receptor antagonist suramin (10 μM; *n* = 6; *N* = 2; *P* < 0.01; Fig. 3E). These results indicate that purinergic signaling participates in PI3K/Akt activation in response to caffeine.

### IRS2 is phosphorylated upon TrkB receptor activation and is required for _CAF_LTP maintenance

**Figure 4:**
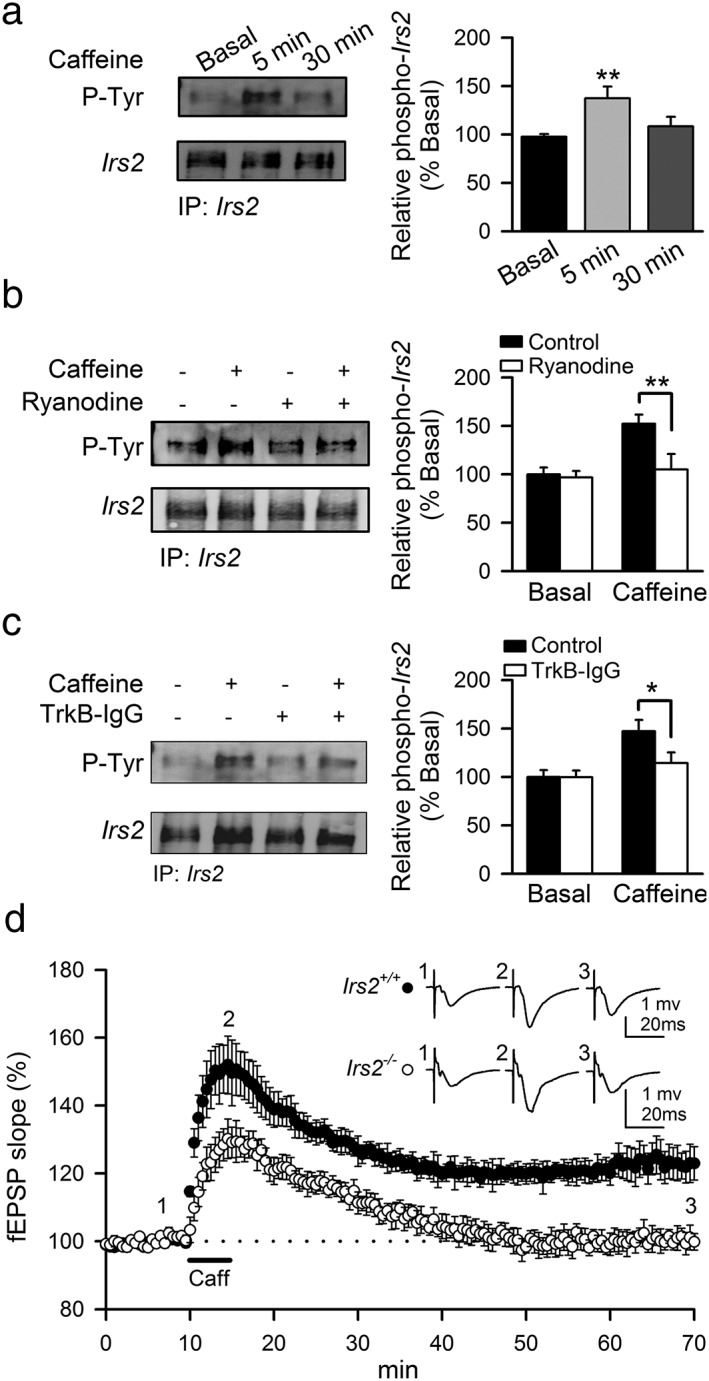
Tyrosine phosphorylation of IRS2 through TrkB signaling pathway is required for _CAF_LTP. (A) Hippocampal slices were collected before (0 min) and after (5 and 30 min) induction LTP with caffeine and IRS2 was immunoprecipitated for Western blot analysis with an anti‐phosphotyrosine antibody (P‐Tyr). Values represent mean ± SEM (*n* = 9; *N* = 3). Significant differences with respect to control were established by one‐way ANOVA followed by Dunnett's *post hoc* test at **P < 0.01. (B and C) Experiments were performed in the presence of 20‐μM ryanodine (B) or 1 µg/ml TrkB‐IgG (C) and hippocampal slices were collected 5 min after induction of LTP with caffeine. IRS2 was immunoprecipitated for Western blotting against P‐Tyr. Values represent mean ± SEM (*n* = 9; *N* = 3 for each treatment). Representative immunoblots are shown. Significant differences were established by two‐way ANOVA followed by Bonferroni's *post hoc* test at *P < 0.05 and **P < 0.01 versus respective caffeine group. (D) Summary of time‐course of mean fEPSPs slope in hippocampal slices from Irs2^+/+^ (filled circles) and Irs2^−/−^ (empty circles) mice in basal conditions and following application of 100‐μM caffeine (*n* = 12; *N* = 4 for each genotype). Traces inset in the plots represent fEPSPs averages recorded during periods indicated by corresponding numbers in the graph

Activation of tyrosine kinase receptors induces the phosphorylation of IRS proteins, enabling them to recruit and activate PI3K through a direct interaction with the p85 catalytic subunit (Myers *et al.* 1992; White 2006). Interestingly, IRS2 signals have been reported to mediate the action of BDNF (Yamada *et al.* 1997) and hippocampal IRS2 is critical for LTP induction upon tetanic stimulation (Martín *et al.* 2012). Because PI3K activity is necessary for _CAF_LTP (Fig. 3B), we hypothesized that the IRS2 adaptor protein may participate in the TrkB‐dependent signaling associated with _CAF_LTP. To address this hypothesis, we evaluated the tyrosine phosphorylation of IRS2 in hippocampal slices in response to caffeine. IRS2 was immunoprecipitated and the immune complexes were subsequently analyzed by Western blotting using an anti‐phosphotyrosine antibody. Interestingly, the tyrosine phosphorylation of IRS2 was significantly increased at 5 min post‐caffeine (137.52 ± 11.98 percent, *n* = 9; *N* = 3, *P* < 0.01), returning to basal levels after 30 min (Fig. 4A). In contrast, caffeine did not promote tyrosine phosphorylation of IRS1 in hippocampal slices (Fig. S2). Consistent with the ability of ryanodine to inhibit caffeine‐induced BDNF release (Fig. 2A), treatment of hippocampal slices with ryanodine (20 μM) completely blocked the tyrosine‐phosphorylation of IRS2 in caffeine‐treated slices (105.05 ± 15.98 percent, *n* = 9; *N* = 3, *P* < 0.01, Fig. 4B). Accordingly, pre‐incubation of hippocampal slices with TrkB‐IgG (1 µg/ml) for 1 hour blocked caffeine‐induced tyrosine phosphorylation of IRS2 at 5 min (114.36 ± 11.01 percent, *n* = 9; *N* = 3, Fig. 4C), suggesting that IRS2 might participate in caffeine‐induced TrkB‐mediated signaling leading to LTP. Importantly, other receptor tyrosine kinases that can phosphorylate IRS proteins such as the IR and IGF‐1R (Sun *et al.* 1995) were not activated as assessed by their tyrosine phosphorylation levels in immunocomplexes obtained from hippocampal slices 5 and 30 min after caffeine application (Fig. S3). Taken together, these results reveal that caffeine evokes an increase of endogenous BDNF secretion that rapidly induces the tyrosine phosphorylation of IRS2 through TrkB signaling pathway.

To further explore the role of IRS2 in _CAF_LTP, we next examined this form of NMDAR‐independent LTP in hippocampus of *Irs2* ^−/−^ mice. First, we assessed basal synaptic transmission by applying isolated stimuli of increasing intensity to hippocampal slices from Irs2^−/−^ and WT mice. The slopes of fEPSP, fiber volleys amplitude and input/output curve in response to individual stimuli were not significantly different between genotypes (*P* > 0.05, *n* = 12; *N* = 4 of each genotype, Fig. S4). PPF ratios of fEPSP slopes at interstimulus intervals ranging from 25 ms to 400 ms were normal in Irs2^−/−^ mice (*n* = 12; *N* = 4 of each genotype, Fig. S4). These findings suggest that the basal synaptic transmission at the pre‐ and post‐synaptic level is normal in Irs2^−/−^ mice. Conversely, caffeine application revealed a marked difference in the ability of hippocampal slices from Irs2^−/−^ mice to support _CAF_LTP, with potentiation of fEPSP being significantly reduced as compared with Irs2^+/+^ slices (*n* = 12; *N* = 4 of each genotype; *P* < 0.01; Fig. 4D). These data support a role for IRS2 signaling in the maintenance of _CAF_LTP.

### IRS2 deficiency impairs activation of PI3K/Akt, which is necessary for expression of _CAF_LTP

**Figure 5:**
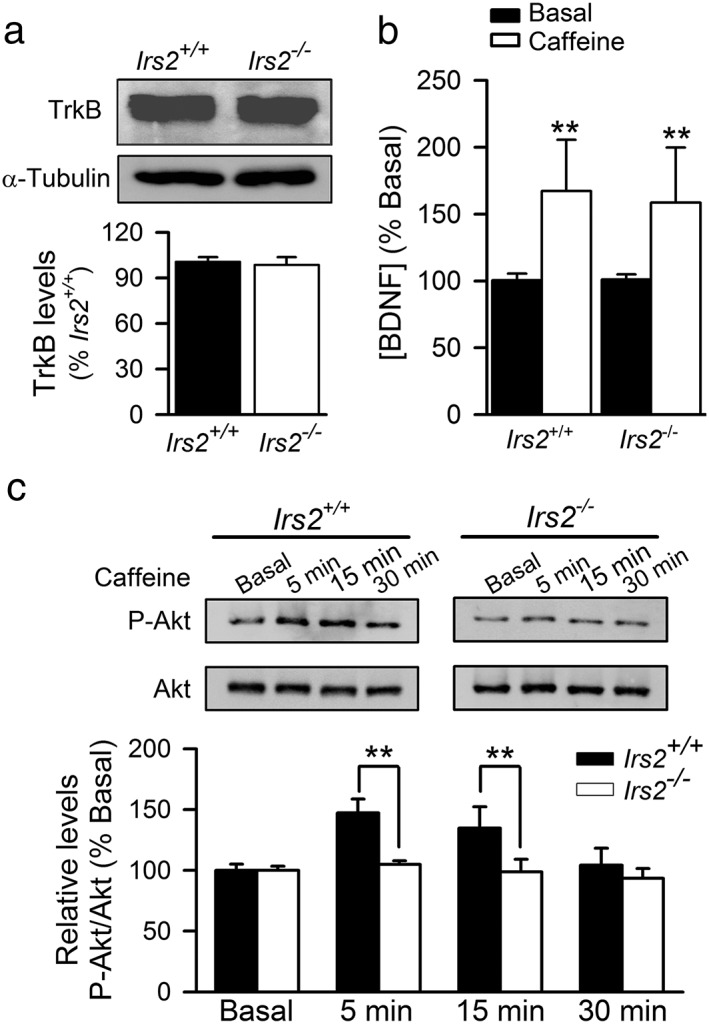
Impaired caffeine‐induced PI3K/Akt pathway signaling in hippocampal slices from Irs2^−/−^ mice. (A) Western blot analysis of hippocampal TrkB levels in Irs2^+/+^ and Irs2^−/−^ mice. Values represent mean ± SEM (*n* = 9; *N* = 4 of each genotype). Representative immunoblots are shown. (B) Effect of caffeine on BDNF release (percent respect to basal) in hippocampal slices from Irs2^+/+^ and Irs2^−/−^ mice (*n* = 9; *N* = 3 of each genotype). (C) Hippocampal slices from Irs2^−/−^ mice were collected before (0 min) and after (5, 15 and 30 min) perfusion with caffeine and processed for Western blot analysis of Akt (Ser473) phosphorylation levels. Values represent mean ± SEM (*n* = 12; *N* = 4 of each genotype). Representative immunoblots are shown. Significant differences were established by two‐way ANOVA followed by Bonferroni's *post hoc* test at **P < 0.01 versus respective caffeine group

We next analyzed the mechanisms underlying the inability of hippocampal slices from Irs2^−/−^ mice to support _CAF_LTP. The impairment of _CAF_LTP in Irs2^−/−^ mice could not be explained by altered levels of TrkB receptors (*n* = 9; *N* = 4 of each genotype; Fig. 5A) or changes in caffeine‐induced BDNF secretion (*n* = 9; *N* = 3 of each genotype; Fig. 5B) compared with wild‐type mice. Therefore, we analyzed the effect of caffeine on Akt phosphorylation in Irs2^−/−^ hippocampal slices. In contrast to the effect on wild‐type mice (*n* = 12; *N* = 4; Fig. 5C), caffeine stimulation failed to promote Akt phosphorylation in hippocampal slices from Irs2^−/−^ mice, at either 5 (104.9 ± 2.91 percent) or 15 (98.7± 10.42 percent; *n* = 12; *N* = 4; Fig. 5C) min, revealing a possible mechanism for the impairment of _CAF_LTP induction in Irs2 null mice. Collectively, our data indicate that _CAF_LTP is mediated by BDNF release and subsequently, by the TrkB–IRS2–PI3K/Akt signaling pathway in postsynaptic neurons.

## Discussion

Several studies indicate that caffeine may act as a cognitive enhancer (Lieberman *et al.* 1987; Durlach 1998; Angelucci *et al.* 1999) and recent reports confirm that this substance improves consolidation of long‐term memory in humans (Borota *et al.* 2014). However, the indirect action of caffeine on arousal, mood and concentration contributes significantly to its cognitive benefits (Nehlig 2010). Because synaptic plasticity is a key component of the learning and memory machinery, we investigated whether caffeine modulates hippocampal LTP, a compelling cellular model of memory. Here we show that caffeine, at moderate doses of human consumption, induces a form of LTP that is dependent on Ca^2+^ release from ryanodine‐sensitive stores. Moreover, this LTP does not require postsynaptic NMDAR activation or GABA_A_‐mediated inhibitory synaptic transmission, in agreement with previous findings (Martín & Buño 2003). Furthermore, _CAF_LTP was characterized by a significant reduction of PPF during perfusion with caffeine, but no changes were observed until 60 min of LTP. Because caffeine acts by antagonizing A1 adenosine receptors (Fredholm *et al.* 1999) and experimental evidence suggests that blockade of A1 receptors decreases PPF (Costenla *et al.* 1999), it is feasible that presynaptic mechanisms are involved in the induction of this form of LTP, whereas consolidation may occur at the postsynaptic level.

We tested whether our _CAF_LTP protocol shares cellular mechanism with the HFS‐tetanic induced LTP. Our results reveal that the induction of a NMDAR‐independent LTP by caffeine did not preclude the induction of classical NMDAR‐dependent LTP by tetanic stimulation, consistent with previous studies where high doses were tested (Lee *et al.* 1987; Martín & Buño 2003). Considering the dose employed in the current study and that caffeine was not present when HFS was elicited, we did not expect any alterations of the levels of post‐tetanic potentiation as was observed by other authors (Lee *et al.* 1987). Interestingly, our results are compatible with other studies where the application of BDNF prior to a tetanic stimulation did not prevent the enhancement of the fEPSPs that are associated with NMDA‐independent LTP (Kang & Schuman 1995), supporting the fact that classical NMDAR‐dependent and caffeine/BDNF NMDAR‐independent LTP rely on different cellular mechanisms.

Various intercellular signaling molecules can trigger lasting changes in the ability of synapses to express plasticity and BDNF‐mediated activation of the TrkB pathway has been implicated in this process. In particular, the neuronal activity‐dependent secretion of BDNF plays a fundamental role in the classical LTP induced by electrical stimulation protocols (Gärtner & Staiger 2002; Rex *et al.* 2007). Interestingly, caffeine increases BDNF levels (Costa *et al.* 2008; Sallaberry *et al.* 2013) and BDNF can induce NMDAR‐independent LTP (Kang & Schuman 1995). Our present results provide the first experimental demonstration that caffeine, at moderate to high concentration, evokes an increase of calcium and neuronal activity‐dependent BDNF secretion during _CAF_LTP in the hippocampus. Moreover, our data suggest a novel mechanism whereby an NMDAR‐independent LTP is maintained through a TrkB‐mediated process.

Several observations from our study support the hypothesis that this form of LTP is mediated by BDNF‐activated TrkB signaling. First, caffeine caused a significant increase in BDNF levels in the perfusion medium, which was abolished by TTX and ryanodine. In addition, disruption of BDNF function by a blocking anti‐TrkB antibody (TrkB‐IgG) significantly reduce _CAF_LTP. Moreover, hippocampal slices from BDNF heterozygous mice displayed a significant decline of _CAF_LTP compared with WT. Previous experimental findings indicate that BDNF may be released at both pre‐ and postsynaptic levels (Lessmann & Brigadski 2009; Matsuda *et al.* 2009; Edelmann *et al.* 2014) and it is known that BDNF can significantly affect PPF (Kang & Schuman 1995). In our experiments, caffeine perfusion induced a significant change in PPF response suggesting an enhancement of the neurotransmitter release. Hence, it is plausible that BDNF is released at the presynaptic level during _CAF_LTP induction. However, based on the present findings, we cannot exclude the possibility that BDNF is released from other sources.

Once BDNF is released into the extracellular environment, it exerts its actions through the TrkB receptor, whose activation triggers three main intracellular signaling cascades: the MAPK pathway, the PI3K/Akt pathway and the PLCγ–Ca^2+^ pathway (Kaplan & Miller 2000). Our data has revealed that inhibition of the PI3K/Akt pathway completely abolished the maintenance of _CAF_LTP, while inhibition of PLCγ or MAPK was ineffective, indicating that activation of PI3K/Akt signaling is necessary to consolidate this form of LTP. In fact, the PI3K/Akt signaling cascade plays an important role in long‐term synaptic plasticity and memory in many brain regions, such as the hippocampus (Sanna *et al.* 2002; Opazo *et al.* 2003) and medial prefrontal cortex (Sui *et al.* 2008). In addition, PI3K is associated with adaptor and scaffolding proteins in the postsynaptic density (Brazil *et al.* 2002) and is implicated in the insertion of AMPA receptors into the membrane (Man *et al.* 2003). In keeping with these reports, our findings establish that induction of LTP was coincident with activation of Akt and that PI3K/Akt signaling is essential to the maintenance of _CAF_LTP. In addition, we show that activation of PI3K/Akt signaling by caffeine is regulated by A1 and P2 purinergic receptors, in agreement with previous reports suggesting that purinergic receptors mediate _CAF_LTP (Martín & Buño 2003).

PI3K does not interact directly with Trk receptors. Adaptor proteins such as GRB‐associated binder‐1, IRS1 and IRS2 are recruited to activate Trk receptors and their phosphorylation leads to downstream activation of PI3K/Akt signaling (Holgado‐Madruga *et al.* 1997; Yamada *et al.* 1997; White 2006). In the present work, we show that caffeine rapidly induces the tyrosine phosphorylation of IRS2, but not IRS1, and that treatment of hippocampal slices with ryanodine blocks caffeine‐induced IRS2 phosphorylation, indicating that release of Ca^2+^ from intracellular ryanodine‐sensitive stores is necessary for the activation of PI3K/Akt signaling in postsynaptic neurons. Although IR and IGF‐1R can also induce IRS phosphorylation (Sun *et al.* 1995), we did not detect their activation in caffeine‐treated hippocampal slices. On the other hand, TrkB‐IgG blocked caffeine‐induced tyrosine phosphorylation of IRS2, further demonstrating the specific involvement of TrkB activation on the downstream signaling that supports _CAF_LTP. In line with this finding, application of caffeine to hippocampal slices from Irs2^−/−^ mice did not induce LTP, indicating that this adaptor protein is required for _CAF_LTP maintenance. In agreement, our results showed that the absence of IRS2 prevents activation of Akt during induction of _CAF_LTP in hippocampal slices, revealing a possible mechanism for the observed impairment of _CAF_LTP induction in Irs2 null animals. This observation is consistent with published studies demonstrating that insulin‐mediated activation of Akt signaling is impaired in Irs2^−/−^ mice (Withers *et al.* 1998; Burks *et al.* 2000). Also in line with our findings that IRS2 is required for _CAF_LTP, IRS2 deficient mice have impaired hippocampal synaptic plasticity in other paradigms (Costello *et al.* 2012; Martín *et al.* 2012). Thus, we conclude that IRS2 is necessary to mediate PI3K‐dependent pathways leading to _CAF_LTP.

It is assumed that at the concentrations typically consumed by humans caffeine acts as CNS stimulant mainly by acting as a competitive antagonist of A_1_ and A_2A_ adenosine receptors (Fredholm *et al.* 1999; Chen *et al.* 2010). Here, we propose an additional complementary mechanism that may explain the effects of caffeine in a cellular and molecular model of learning and memory. Caffeine antagonizes presynaptic A_1_ adenosine receptors and mobilizes Ca^2+^ from ryanodine‐sensitive intracellular stores (Martín & Buño 2003). Our present results suggest a novel model where this rise of intracellular Ca^2+^ may trigger BDNF secretion close to the synaptic cleft where it activates TrkB, followed by phosphorylation of IRS2 on the appropriate residues to recruit PI3K/Akt signaling pathway. Thus, we also conclude that IRS2 expression and/or function in the brain is required for physiological modulation of different forms of synaptic plasticity.

## Author Contribution

EDM, MF and DB were responsible for the study concept and design. CLP, JJB, MF, AZM and AS performed the experiments, data analysis and interpreted the results. MGL contribute to ELISA experiment. EDM, DB and EPN, assisted with data analysis and interpretation of findings. EDM drafted the manuscript. DB, MF, EPN and AS provided critical revision of the manuscript for important intellectual content. All authors critically reviewed content and approved final version for publication.

## Supporting information


**Supplementary Figure 1**. **Basal synaptic transmission and PPF at CA1 are not modified by ryanodine**. (A) fEPSP slopes were comparable in Control (filled circle) and in the presence of 20‐μM ryanodine (Ryan; empty circle) for a given range of stimulus intensities. (B) Fiber volley amplitudes are similar in Control (filled circle) and in the presence of 20‐μM ryanodine (empty circle) for a given range of stimulus intensities. (C) Input/output relationships for Control (filled circle) and in the presence of 20‐μM ryanodine (empty circle). (D) Paired‐pulse facilitation of fEPSPs was similar in both Control (filled circle) and in the presence of 20‐μM ryanodine (empty circle) The mean slope of the paired EPSP is plotted against interpulse interval. Data are presented as mean ± SEM (*n* = 7; *N* = 1 for all figures).
**Supplementary Figure 2**. **Caffeine does not promote the tyrosine phosphorylation of IRS1**. Hippocampal slices were collected before (0 min) and after (5 min) LTP induction with caffeine. IRS1 was immunoprecipitated and analyzed by Western blot analysis with an anti‐phosphotyrosine antibody (P‐Tyr). Values represent mean ± SEM (*n* = 10; *N* = 2).
**Supplementary Figure 3**. **Effect of caffeine on the activation of IR and IGF‐1R in the hippocampus**. Hippocampal slices were collected before (0 min) and after (5 and 30 min) perfusion with caffeine and processed for immunoprecipitation of IR (A) or IGF‐1R (B) and Western blotting analysis of phosphotyrosine (P‐Tyr) levels. Values represent mean ± SEM (*n* = 9; *N* = 3). Representative immunoblots are shown.
**Supplementary Figure 4**. **Basal synaptic transmission and PPF at CA1 synapses are preserved in *Irs2*** ^−/−^ **mice**. (A) fEPSP slopes are comparable in Irs2^+/+^ (filled circle) and Irs2^−/−^ (open circle) for a given range of stimulus intensities. (B) Fiber volley amplitudes are similar in Irs2^+/+^ (filled circle) and Irs2^−/−^ (open circle) for a given range of stimulus intensities. (C) Input/output relationships for wild type (filled circle) and transgenic (open circle) mice. (D) Paired‐pulse facilitation of fEPSPs was similar in both Irs2^+/+^ (filled circle) and Irs2^−/−^ (open circle) mice. The mean slope of the paired EPSP is plotted against interpulse interval. Data are presented as mean ± SEM (*n* = 9; *N* = 4 for all figures).

Supporting info item

Supporting info item

Supporting info item

Supporting info item

Supporting info item
