# Supplementary figures and images for: Caffeine‐mediated BDNF release regulates long‐term synaptic plasticity through activation of IRS2 signaling
Source: Addict Biol. 2016 Jul 25;22(6):1706–18. doi: 10.1111/adb.12433 (PMC5697621; doi:10.1111/adb.12433)

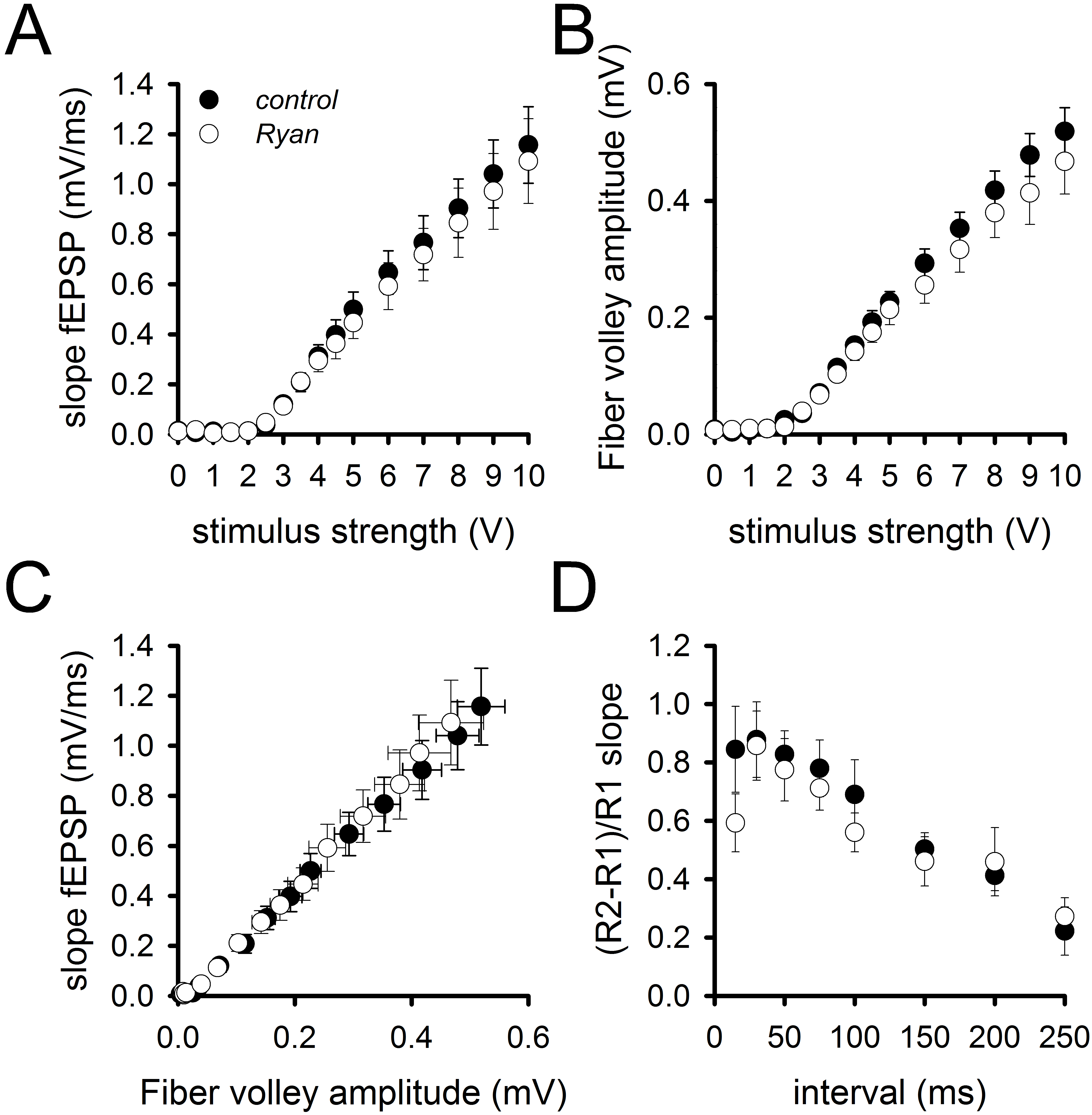

Supplement: Supplementary file 1 — Supporting info item [file ADB-22-1706-s001.TIF]

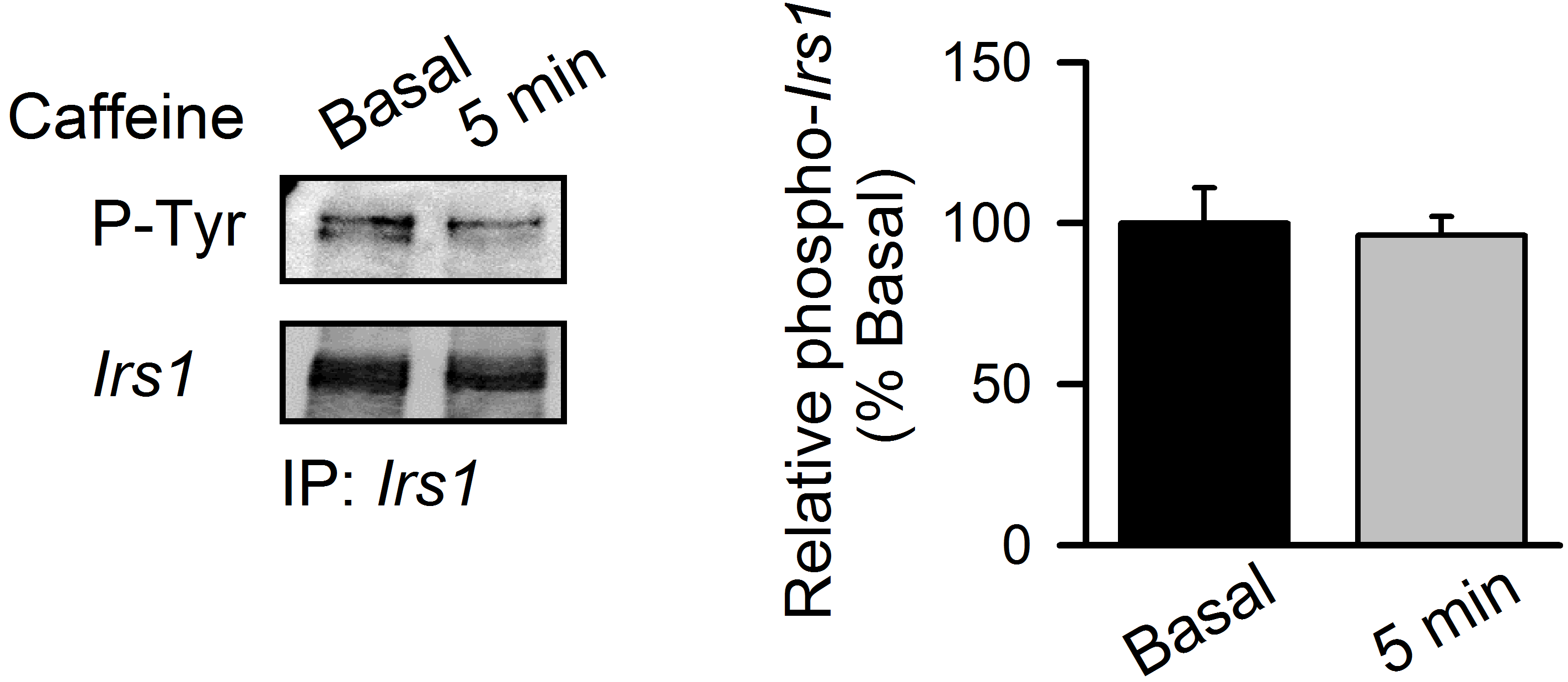

Supplement: Supplementary file 2 — Supporting info item [file ADB-22-1706-s002.TIF]

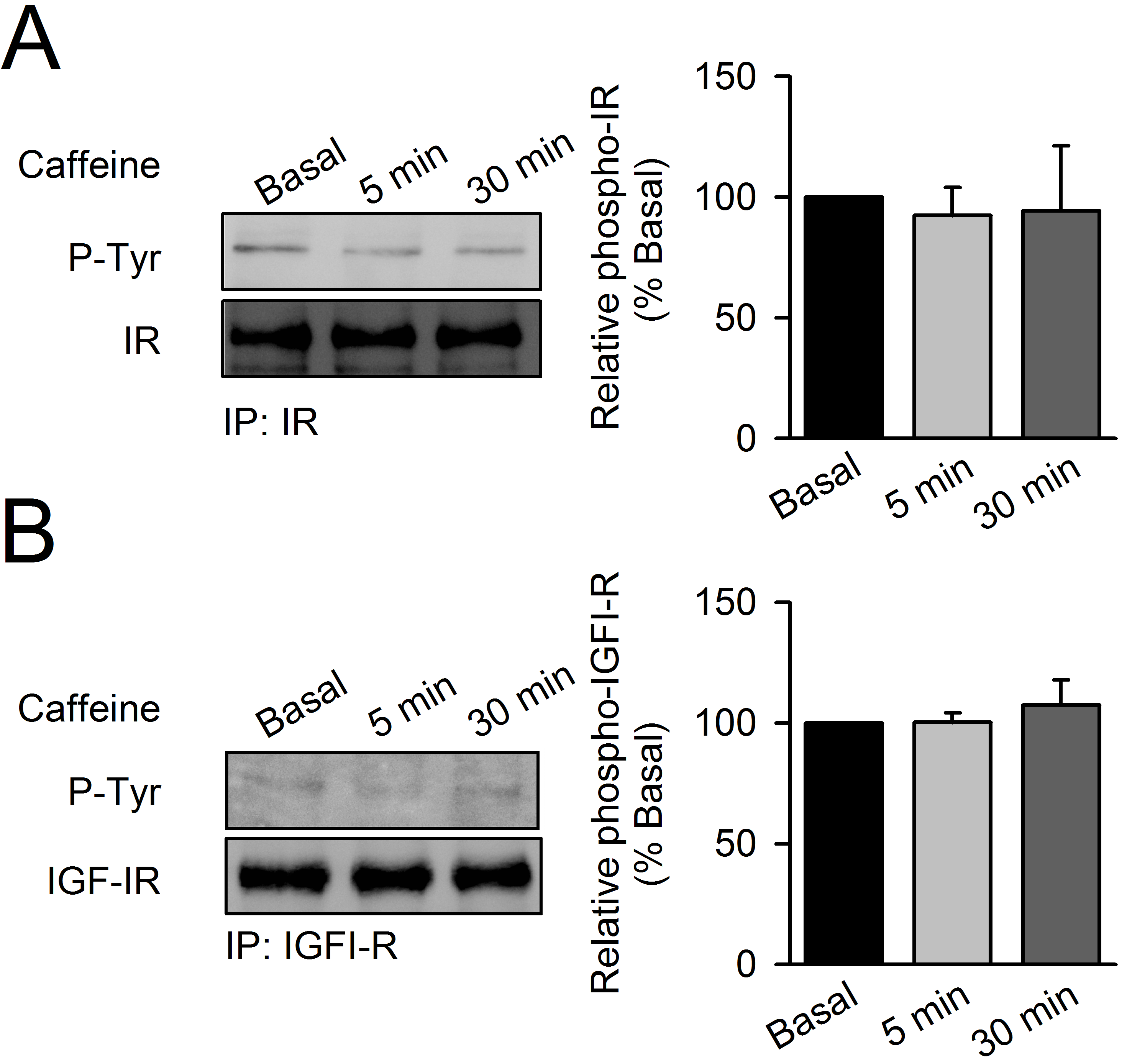

Supplement: Supplementary file 3 — Supporting info item [file ADB-22-1706-s003.TIF]

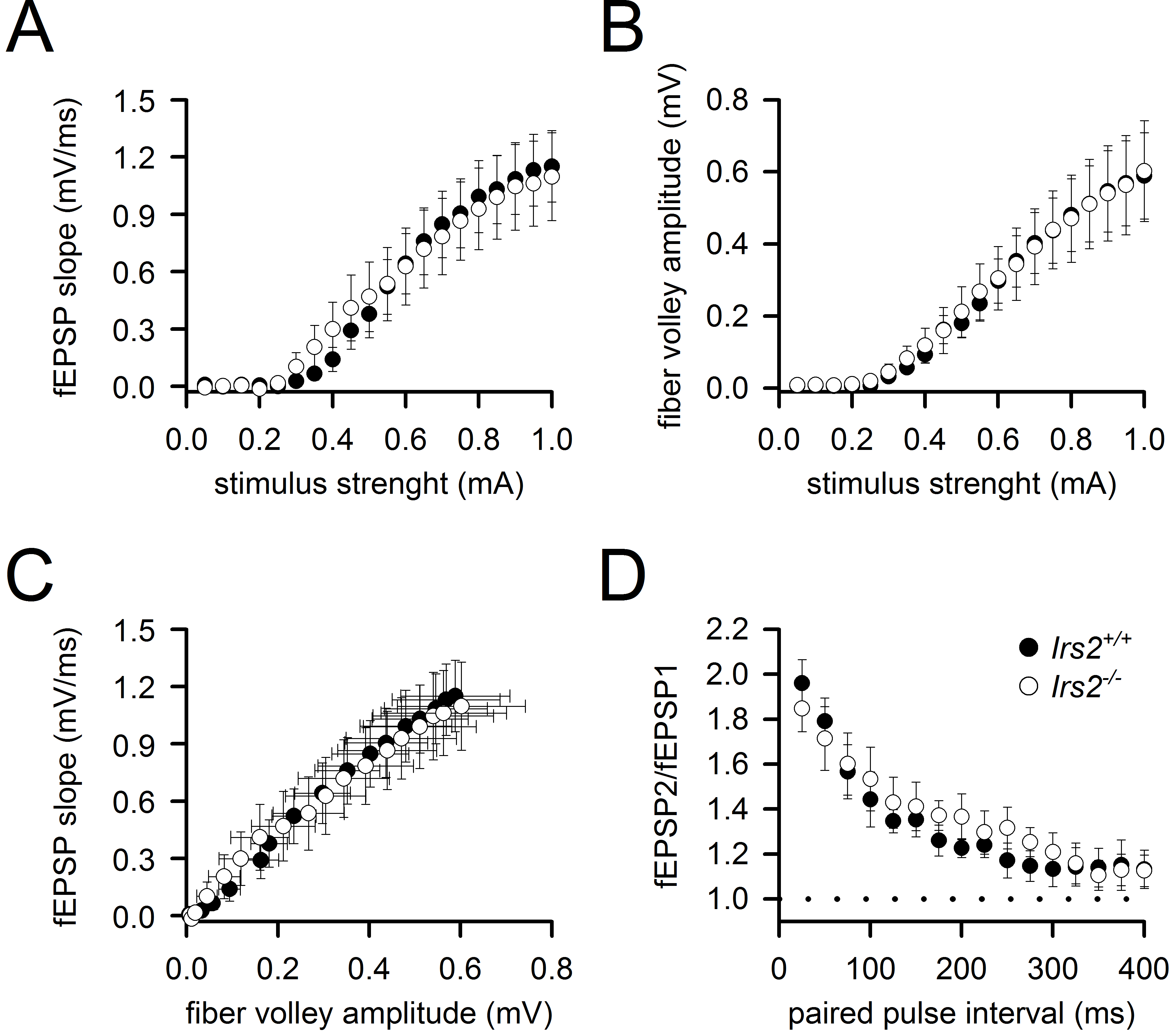

Supplement: Supplementary file 4 — Supporting info item [file ADB-22-1706-s004.TIF]
